# Characteristics of non-exercise activity thermogenesis in male collegiate athletes under real-life conditions

**DOI:** 10.3389/fspor.2024.1326890

**Published:** 2024-02-13

**Authors:** Mika Goshozono, Nozomi Miura, Suguru Torii, Motoko Taguchi

**Affiliations:** ^1^ Graduate School of Sport Sciences, Waseda University, Tokorozawa, Japan; ^2^ Faculty of Sport Sciences, Waseda University, Tokorozawa, Japan

**Keywords:** non-exercise activity thermogenesis, activity intensity, activity time, energy expenditure, energy components, athletes

## Abstract

Athletes experience high total energy expenditure; therefore, it is important to understand the characteristics of the components contributing to this expenditure. To date, few studies have examined particularly the volume and activity intensity of non-exercise activity thermogenesis (NEAT) in athletes compared to non-athletes under real-life conditions. This study aimed to determine the volume and intensity of NEAT in collegiate athletes. Highly trained Japanese male collegiate athletes (*n* = 21) and healthy sedentary male students (*n* = 12) participated in this study. All measurements were obtained during the athletes' regular training season under real-life conditions. NEAT was calculated using metabolic equivalent (MET) data using an accelerometer. The participants were asked to wear a validated triaxial accelerometer for 7 consecutive days. Physical activity intensity in NEAT was classified into sedentary (1.0–1.5 METs), light (1.6–2.9 METs), moderate (3.0–5.9 METs), and vigorous (≥6 METs) intensity. NEAT was significantly higher in athletes than in non-athletes (821 ± 185 kcal/day vs. 643 ± 164 kcal/day, *p* = 0.009). Although there was no significant difference in NEAT values relative to body weight (BW) between the groups (athletes: 10.5 ± 1.7 kcal/kg BW/day, non-athletes: 10.4 ± 2.2 kcal/kg BW/day, *p* = 0.939), NEAT to BW per hour was significantly higher in athletes than in non-athletes (0.81 ± 0.16 kcal/kg BW/h vs. 0.66 ± 0.12 kcal/kg BW/h, *p* = 0.013). Athletes spent less time in sedentary and light-intensity activities and more time in vigorous-intensity activities than non-athletes (*p* < 0.001, *p* = 0.019, and *p* = 0.030, respectively). Athletes expended more energy on vigorous- and moderate-intensity activities than non-athletes (*p *= 0.009 and *p* = 0.011, respectively). This study suggests that athletes' NEAT relative to BW per day is similar to that of non-athletes, but athletes spend less time on NEAT, which makes them more active in their daily lives when not exercising and sleeping.

## 1 Introduction

Athletes need an energy intake that matches their daily energy expenditure to maintain and improve health and performance (1). However, athletes' total energy expenditure (TEE) is known to be very high at 4,500 kcal (2) and highly variable (3). It is important to clarify the characteristics of TEE components and assess TEE appropriately to ensure proper nutritional management among athletes.

TEE mainly consists of resting energy expenditure (REE), diet-induced thermogenesis (DIT), and activity-induced energy expenditure (AEE), which is further divided into non-exercise activity thermogenesis (NEAT) and exercise energy expenditure (EEE) (4, 5). Previous studies on athletes have examined the amounts of energy expended in REE (6), DIT (7), and EEE (8); however, research on NEAT is extremely limited. DIT is the energy expenditure resulting from food digestion, absorption, and nutrient storage. Although it is the smallest component of TEE, it has been suggested that it may be involved in the development and/or maintenance of obesity (9). NEAT is defined as the energy expenditure required for activities of daily living, including standing, walking, talking, and shopping (10). Many NEAT studies have focused on sedentary adults, particularly those who are overweight or obese. Previous studies have shown that low NEAT levels are associated with obesity (11), while reducing low-intensity activity time and increasing physical activity are effective in preventing obesity and chronic diseases (12). However, very few athletes are obese. NEAT is influenced by various factors, including occupation, urban environment, sex, age, body composition, season, and education (13). Due to the vastly different characteristics of the populations, comparing athletes’ NEAT to that of overweight/obese individuals and older age groups may distort interpretation. Therefore, to clarify the NEAT characteristics of athletes, it is necessary to compare them with participants of the same sex and age group. Although increased NEAT is considered beneficial to health (14), it is not necessarily beneficial for athletes. Athletes need energy intake to match their energy expenditure; however, if the total energy intake (TEI) cannot match the increase in NEAT, the energy balance may become negative. An exercise training intervention study (15) found that participants tended to compensate for increased energy expenditure associated with exercise training by reducing non-training activities and spending the rest of the day on sedentary activities. A meta-analysis on sedentary behavior and physical activity in competitive and recreational athletes (16) revealed that athletes spent significantly more time engaging in sedentary behavior than the general population (time in sedentary behavior; 576 ± 136 min/day vs. 513 ± 105 min/day). Athletes have high EEE, and they may compensate for the increased energy expenditure associated with exercise training by increasing time in sedentary behavior and decreasing NEAT volume in their daily activities. To our knowledge, few studies have examined particularly the volume and activity intensity of NEAT in athletes compared to non-athletes under real-life conditions. Determining the NEAT characteristics of athletes will help suggest the appropriate energy intake for this population.

This study aimed to determine the volume and intensity of NEAT in collegiate athletes.

## 2 Methods

### 2.1 Participants

This cross-sectional study included highly trained Japanese male collegiate athletes (*n* = 21; athletes; age: 19 ± 1 years) and healthy male sedentary students with no exercise habits (*n* = 12; non-athletes; age: 21 ± 2 years) from the same university. The recruited athletes were classified as Tier 3 athletes (17), participating in the national or regional leagues/tournaments, while non-athletes were classified as Tier 0 sedentary individuals with an average weekly training volume of less than 150 min/week. The athletes included 16 football players and 5 lacrosse players. The inclusion criteria were as follows: age 18–25 years, non-smoking status, no use of medications influencing metabolic or reproductive hormones, and absence of diseases or injuries. All measurements were performed during the athletes' regular training season under real-life conditions between October 2022 and January 2023. Before starting the study, all participants received an oral explanation of the study and provided written informed consent. This study was approved by the Ethics Review Committee on Research with Human Subjects of Waseda University and conducted in accordance with the Declaration of Helsinki (2022-306).

### 2.2 Body composition

After overnight fasting, body weight (BW) was measured to the nearest 0.05 kg using an electronic scale (UC-321; A&D Co., Ltd., Tokyo, Japan). Height was measured to the nearest 0.1 cm using a stadiometer (YG-200; Yagami Inc., Tokyo, Japan). Body mass index (BMI) was calculated by dividing BW (kg) by the square of the height (m^2^). The body fat percentage was measured using dual-energy x-ray absorptiometry (DXA) (Horizon A DXA scanner; Hologic Inc., Marlborough, MA, USA). All scans and analyses were conducted by an experienced orthopedic surgeon and analyzed using Hologic software (version. 12.4.3, Hologic Inc.). The mean coefficient of variance (CV) of the measurements was less than 1%. Fat mass was calculated from BW and body fat percentage. Fat-free mass (FFM) was calculated by subtracting fat mass from BW.

### 2.3 Resting energy expenditure

**Figure 1:**
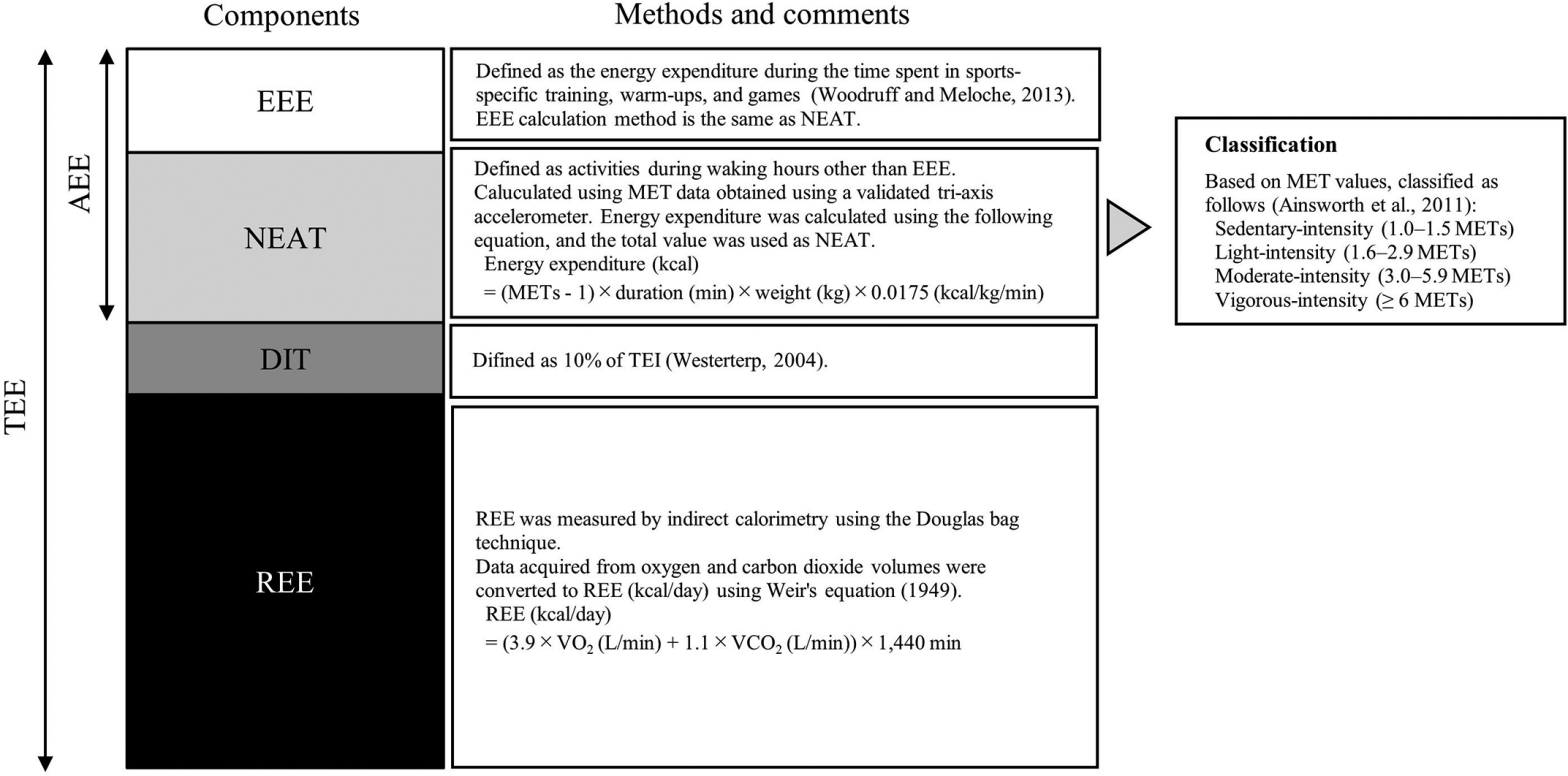
Components of energy expenditure and overview of methods used in calculations. TEE consists of REE, DIT, and AEE; AEE is divided into NEAT and EEE. Based on its intensity, NEAT physical activity is classified into four levels: sedentary (sedentary, 1.0–1.5 METs), light (light, 1.6–2.9 METs), moderate (moderate, 3.0–5.9 METs), and vigorous (vigorous, ≥6 METs). TEE, total energy expenditure; AEE, activity-induced energy expenditure; REE, resting energy expenditure; DIT, diet-induced thermogenesis; NEAT, non-exercise activity thermogenesis; EEE, exercise-energy expenditure; TEI, total energy intake; METs, metabolic equivalents.

 Figure 1 shows the structure and method of the TEE components. REE was measured by indirect calorimetry using the Douglas bag technique. Measurements were performed in the laboratory between 7:00 a.m. and 9:00 a.m. after 10–12 h of fasting, with the exception of drinking water uptake. On the day of the measurements, the participants traveled leisurely from their homes to the laboratory and lay in a supine position in a quiet room maintained at approximately 22°C–24°C for at least 30 min until their heart rate reached a resting state. Two 10-min samples of expired gas were collected in Douglas bags. Expired air volume was measured using a dry gas volume meter (DC-5A; Shinagawa, Tokyo, Japan). Oxygen consumption and carbon dioxide production were analyzed using a gas analyzer (AE100i; Minato 175 Medical Science Co., Ltd., Osaka, Japan). Data acquired from oxygen and carbon dioxide volumes were converted to REE (kcal/day) using Weir's equation (18). The measurements were repeated until the CV of the REE was less than 5%, and the mean values of the two samples were used for the analysis (CV = 1.8%).

### 2.4 Diet-induced thermogenesis and total energy intake

The DIT values differ for each nutrient; however, for healthy participants consuming a mixed diet, the DIT represents approximately 10% of the total energy intake over 24 h (9). Therefore, the DIT was calculated as 10% of the TEI. The participants were instructed to record and photograph all foods and beverages consumed and to weigh food using a kitchen scale for 7 consecutive days to assess TEI. The participants were then interviewed about the foods consumed, and photographs were recorded by a sports dietitian, who was one of the authors (MG). TEI was calculated using nutritional analysis software Wellness 21 (version 2.86; Top Business System, Okayama, Japan) based on the Standard Tables of Food Composition in Japan 2020 (Eighth Revised Edition).

### 2.5 Non-exercise activity thermogenesis and exercise energy expenditure

EEE is defined as the energy expended during the time spent in sports-specific training, warm-ups, and games (19), while other waking-hour activities were included in NEAT. NEAT and EEE were calculated using metabolic equivalent (MET) data obtained using an accelerometer. In this study, the participants were asked to wear a validated triaxial accelerometer (Active Style Pro HJA-750C; 23 g, 40 × 52 × 12 mm; Omron, Kyoto, Japan) at the waist for the same 7 consecutive days as the TEI recording, except while sleeping and bathing. This accelerometer provides measurements of acceleration signals in the anteroposterior (*x*-axis), mediolateral (*y*-axis), and vertical (*z*-axis) directions. The validity of the accelerometer's MET estimation was confirmed using the Douglas bag method (20). The accelerometer is reported to have a high accuracy (*r* = 0.88), with the TEE measured using the double-labeled water method under real-life conditions (21). Participants were instructed to record all activities, including times and durations of non-wear periods, in their activity diaries to account for missing data on activities for which wearing the accelerometer was not possible, such as during bathing. Results were only included in the analysis when participants wore the accelerometer for more than 90% of their awaking time. Non-wear period activities were assigned MET values based on the compendium of physical activities (22). The time of the day (1,440 min) was divided into three parts based on activity diaries: sleep, NEAT, and EEE. MET data collected in 10-s epochs using an accelerometer were used to determine the duration (min) of each MET for NEAT and EEE. One MET was defined as oxygen consumption of 3.5 mL/kg/min (23), converted to 0.0175 kcal/kg/min. Energy expenditure was calculated by subtracting 1.0 MET (REE) from the collected MET data using the following formula:

Energyexpenditure(kcal)=(METs–1)×duration(min)×bodyweight(kg)×0.0175(kcal/kg/min)

The sum of the energy expenditure during the NEAT period was defined as the NEAT (kcal/day), and the sum of the energy expenditure during the EEE period was defined as the EEE (kcal/day).

Physical activity intensity was classified into four levels: sedentary (1.0–1.5 METs), light (1.6–2.9 METs), moderate (3.0–5.9 METs), and vigorous (≥6 METs) (22), and the time spent at each intensity level was determined. The relative percentage of NEAT at each intensity level was calculated.

### 2.6 Total energy expenditure and relative percentage of TEE

TEE was calculated by summing the REE, DIT, NEAT, and EEE values. REE, DIT, NEAT, and EEE were expressed as percentages of TEE.

### 2.7 Statistical analysis

IBM SPSS Statistics (version 28.0, IBM Japan, Tokyo, Japan) was used for the statistical analyses. All data were assessed for normality using the Shapiro–Wilk test before statistical analyses were performed. Data are presented as the mean ± standard deviation (SD).

Student's *t*-test was used to compare differences between groups for normally distributed data. For non-normally distributed data [body fat, fat mass, BMI, DIT (kcal/kg BW), NEAT (kcal/kg BW/h), NEAT sedentary (min), NEAT vigorous (min), and NEAT vigorous (%), EEE (kcal), EEE (kcal/kg BW), EEE (%), and EEE (min)], the Mann–Whitney *U*-test was used to compare differences between groups. In all analyses, statistical significance was set at *p* < 0.05. The effect sizes (ES) were calculated using Cohen's *d*, with effect size threshold values of trivial (<0.2), small (0.2–0.5), moderate (0.5–0.8), and large (>0.8).

## 3 Results

**Figure 2:**
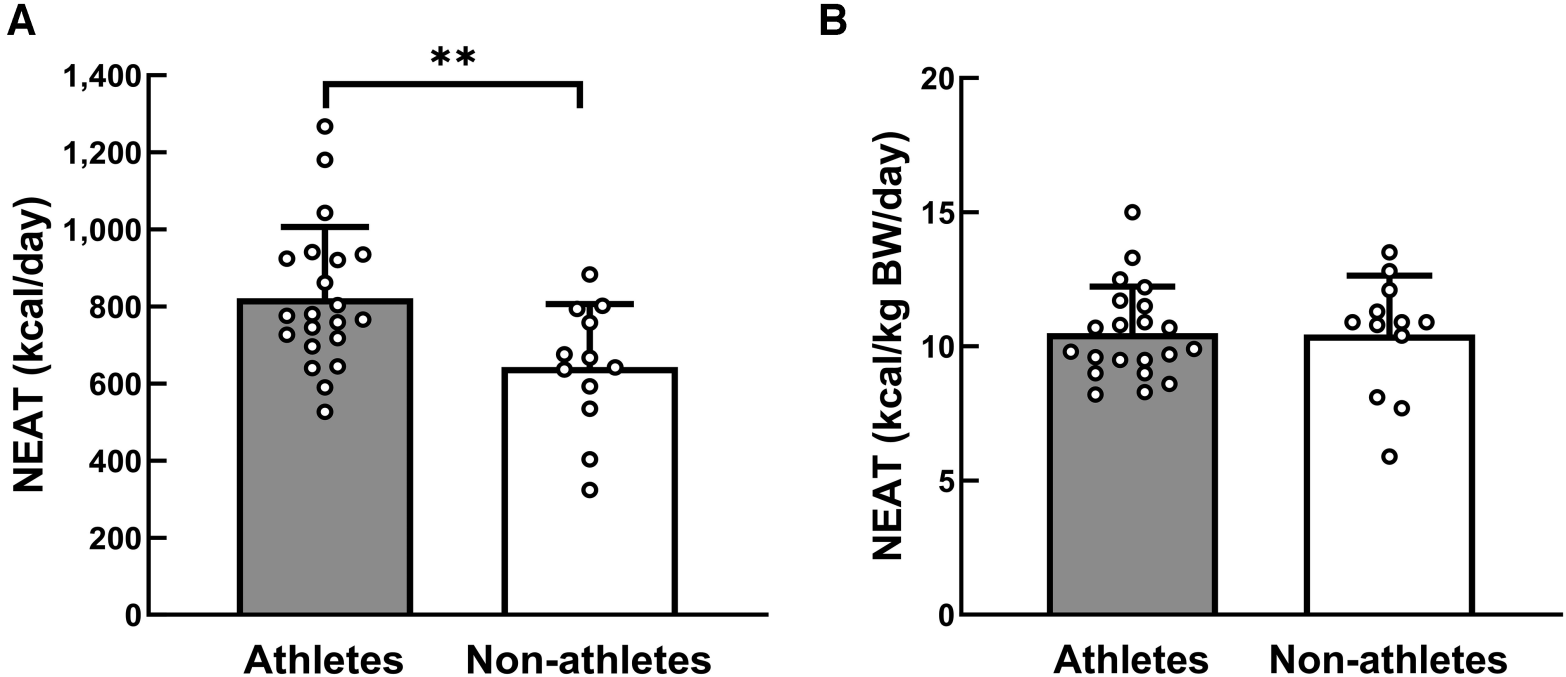
Distribution of NEAT per day (**A**) and per kg of BW per day (**B**). Circles represent individual data. **Significantly different between the two groups, *p* < 0.01. NEAT, non-exercise activity thermogenesis; BW, body weight.

**Figure 3:**
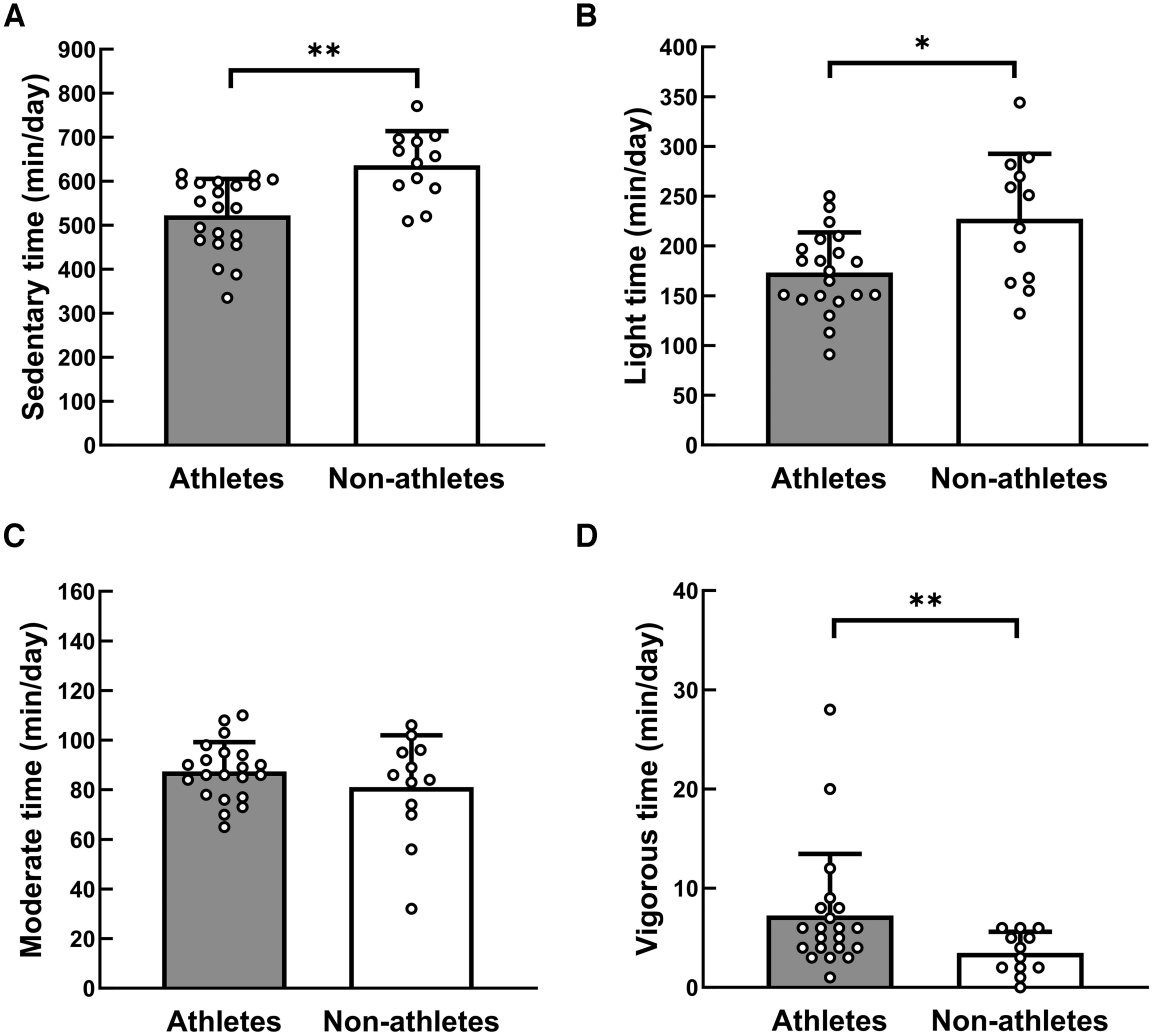
Comparison of the time spent at different intensity levels within NEAT. Physical activity intensity was classified into four levels: (**A**) sedentary (1.0–1.5 METs), (**B**) light (1.6–2.9 METs), (**C**) moderate (3.0–5.9 METs), and (**D**) vigorous ( ≥6 METs) (22). Circles indicate the individual data. *Significantly different between the two groups, *p* < 0.05, **Significantly different between the two groups, *p* < 0.01. NEAT, non-exercise activity thermogenesis.

**Figure 4:**
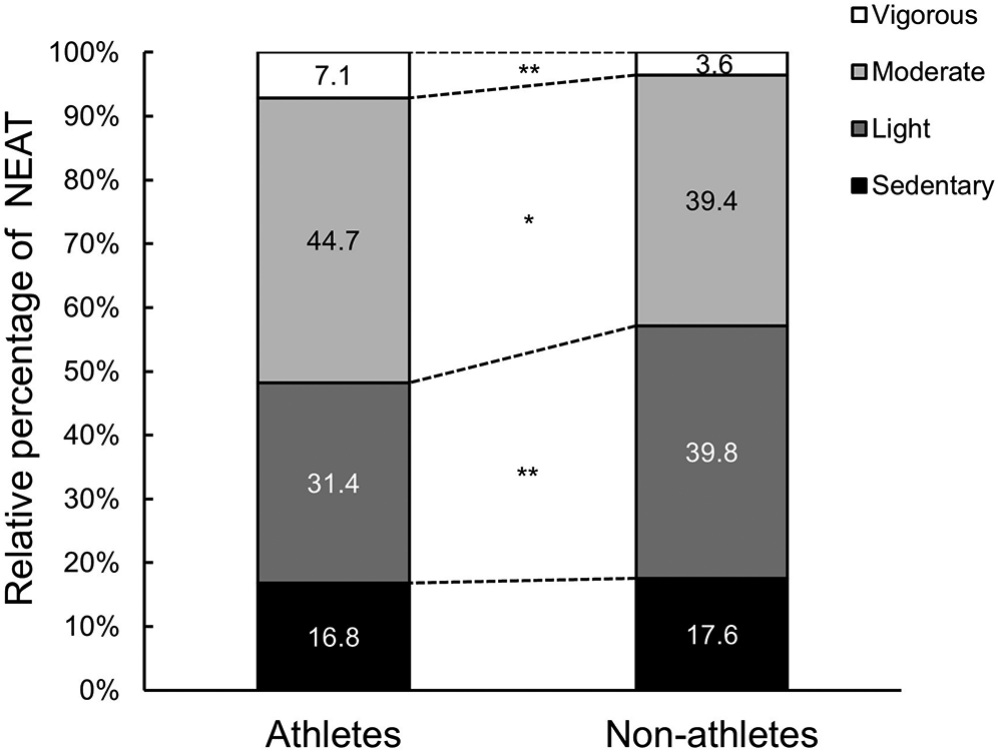
Comparison of the mean relative percentages of each activity within NEAT. *Significantly different from the athletes, *p* < 0.05, **Significantly different from the athletes, *p* < 0.01. NEAT, non-exercise activity thermogenesis.

All 21 athletes and 12 non-athletes enrolled in this study completed the data collection. Table 1 presents the characteristics of the participants. BW, BMI, and FFM were higher in athletes than non-athletes. Table 2 presents a comparison of TEI, TEE, and TEE components. NEAT per day was higher in athletes than their counterparts (*p* = 0.009, *d* = 1.01). In contrast, there was no significant difference in NEAT relative to BW between groups (*p* = 0.939, *d* = 0.03). NEAT to BW per hour was higher in athletes than non-athletes (0.81 ± 0.16 kcal/kg BW/h vs. 0.66 ± 0.12 kcal/kg BW/h, *p* = 0.013, *d* = 1.07). Figure 2 shows that NEAT was widely distributed in both groups. Table 3 presents the time spent sleeping, NEAT, and EEE. There was no significant difference in sleep duration between the groups; the athletes spent more time on EEE and less time on NEAT during the day. Figure 3 shows the average daily time spent on sedentary (A), light- (B), moderate- (C), and vigorous-intensity activities (D) within the NEAT. The athletes spent less time on sedentary activities (athletes: 522 ± 83 min/day, non-athletes: 636 ± 77 min/day, *p* < 0.001, *d* = 1.41) and light-intensity activities (athletes: 173 ± 40 min/day, non-athletes: 227 ± 65 min/day, *p* = 0.019, *d* = 1.07) and more time on vigorous-intensity activities (athletes: 7 ± 6 min/day, non-athletes: 4 ± 2 min/day, *p* = 0.030, *d* = 0.71). Figure 4 shows the average percentage of energy expenditure in the physical activity intensity category within NEAT. The athletes expended more energy in vigorous- and moderate-intensity activities and less energy in light-intensity activities.

**Table 1:** Characteristics of the participants.

	Athletes (*n* = 21)	Non-athletes (*n* = 12)	*p*-Value	ES
Height (cm)	175.0 ± 4.9	170.7 ± 7.7	0.059	0.70
Body weight (kg)	78.1 ± 10.8	61.6 ± 9.6	<0.001	1.58
BMI (kg/m^2^)	25.5 ± 3.2	21.1 ± 2.8	<0.001	1.41
Body fat (%)	14.2 ± 3.8	15.5 ± 4.1	0.449	0.32
Fat mass (kg)	11.4 ± 4.7	9.6 ± 3.4	0.365	0.42
FFM (kg)	66.6 ± 6.6	52.0 ± 7.6	<0.001	2.11

All data are reported as mean ± SD. BMI, body mass index; FFM, fat-free mass; ES, effect size.

**Table 2:** Comparison of TEI, TEE, and TEE components per day.

	Athletes (*n* = 21)	Non-athletes (*n* = 12)	*p*-Value	ES
TEI (kcal)	3,499 ± 676	2,005 ± 351	<0.001	2.57
TEE (kcal)	3,491 ± 423	2,210 ± 313	<0.001	3.30
TEE components				
REE (kcal)	1,815 ± 169	1,367 ± 177	<0.001	2.61
(kcal/kg BW)	23.5 ± 2.4	22.3 ± 2.3	0.195	0.48
DIT (kcal)	350 ± 68	200 ± 35	<0.001	2.57
(kcal/kg BW)	4.5 ± 0.9	3.3 ± 0.8	<0.001	1.41
NEAT (kcal)	821 ± 185	643 ± 164	0.009	1.01
(kcal/kg BW)	10.5 ± 1.7	10.4 ± 2.2	0.939	0.03
EEE (kcal)	504 ± 33	0 ± 0	<0.001	4.17
(kcal/kg BW)	6.5 ± 1.8	0 ± 0	<0.001	4.49
Relative percentage of TEE				
REE (%)	52.3 ± 4.7	62.1 ± 4.7	<0.001	2.06
DIT (%)	10.0 ± 1.5	9.1 ± 1.6	0.140	0.55
NEAT (%)	23.4 ± 3.3	28.8 ± 4.2	<0.001	1.43
EEE (%)	14.3 ± 3.0	0 ± 0	<0.001	5.84

All data are reported as mean ± SD. TEE, total energy expenditure; REE, resting energy expenditure; DIT, diet-induced thermogenesis; NEAT, non-exercise energy expenditure; EEE, exercise energy expenditure; BW, body weight; ES, effect size.

**Table 3:** Time spent on different activities over the day.

	Athletes (*n* = 21)	Non-athletes (*n* = 12)	*p*-Value	ES
Sleep (min)	469 ± 66	492 ± 67	0.356	0.34
NEAT (min)	790 ± 79	949 ± 67	<0.001	2.11
EEE (min)	179 ± 42	0 ± 0	<0.001	5.30

All data are reported as mean ± SD. NEAT, non-exercise energy expenditure; EEE, exercise energy expenditure; ES, effect size.

## 4 Discussion

The present study was designed to characterize the NEAT in collegiate athletes. The primary finding of this study revealed that athletes exhibited a higher NEAT per day than non-athletes. Although there was no significant difference in NEAT relative to BW between the groups, NEAT to BW per hour was significantly higher in athletes than non-athletes. Athletes spent more time on vigorous-intensity activities and less time on sedentary and light-intensity activities. Athletes expended more energy during moderate- and vigorous-intensity activities. Therefore, our findings suggest that athletes are more active than non-athletes in their daily lives, excluding exercise and sleep. To the best of our knowledge, this is the first study to examine NEAT characteristics in male collegiate athletes in terms of volume and activity intensity under real-life conditions.

NEAT is the energy expenditure for activities of daily living (10), showing wide variations depending on biological and environmental factors (11). To date, no consensus has been reached regarding high or low NEAT levels in athletes. Our study showed that the NEAT per day was higher in athletes than in non-athletes (Table 2). The sample size, significance level (*p* < 0.05), and effect size for this study resulted in a *post-hoc* power of 77% for NEAT (kcal), calculated using G*Power 3.1.9.7. Energy expenditure was determined by body size, activity intensity, and activity duration. In this study, there was no significant difference in NEAT relative to BW between the groups. One factor contributing to the difference in NEAT per day between the groups was the difference in body size. However, the athletes' NEAT time was 790 ± 79 min, approximately 160 min shorter than the non-athletes (Table 3). Furthermore, NEAT to BW per hour was significantly higher in athletes than non-athletes. These findings were similar to the previous study; the time-adjusted NEAT differed between exercise and non-exercise conditions (24). Therefore, the intensity and duration of NEAT activity were examined in detail in this study. A meta-analysis on sedentary behavior and physical activity in athletes (16) showed that athletes were significantly more inactive than the general population (time in sedentary behavior; 576 ± 136 min/day vs. 513 ± 105 min/day). Alméras et al. (25) found no significant differences in daily energy expenditure or physical activity patterns during non-exercise periods between cross-country skiers and sedentary men. The results of these previous studies are inconsistent with our results. We found that much of the athletes' waking non-training hours were spent performing sedentary activities (522 ± 83 min/day) requiring less than 1.5 METs, such as sitting, lying down, studying, or taking classes. However, the athletes’ sedentary-intensity activity time was significantly less than the non-athletes' time (636 ± 77 min/day). This difference from previous studies can be explained by the characteristics of the participants. This study was conducted on collegiate students, whereas most previous studies have been conducted on elite and older athletes. The collegiate athletes in this study exercised 179 ± 42 min/day and were engaged in moderate- to vigorous-intensity activities in their daily lives, such as running or walking to catch the train to the training place and working part-time. Non-athletes spent more time in sedentary or light-intensity activities such as sedentary computer work, studying, and watching videos. Therefore, it is likely that athletes spend less time on sedentary or low-intensity activities and more energy on moderate- or vigorous-intensity activities. There was no significant difference in sleep duration between athletes and non-athletes. Athletes expended more energy in moderate- to vigorous-intensity activities, suggesting that athletes were more active than non-athletes in their daily lives, excluding exercise and sleep.

The NEAT in the present study (821 ± 185 kcal/day) was similar to that of male distance runners, cyclists, and triathletes under real-life conditions [819 (482–1,648) kcal/day] (26). However, the average NEAT among athletes in this study was higher than male collegiate soccer players under real-life conditions (456 ± 199 kcal/day) (27). NEAT exhibits wide distribution among athletes, ranging from 456 kcal/day (27) to 1,661 kcal/day (28), and a consistent opinion on NEAT volume has not yet been reached. The NEAT result of LEE et al. (27) relative to BW was approximately 7.5 kcal/kg BW/day, which is smaller than the value in the present study. The variations in NEAT between the study by Lee et al. (27) and this study were due to differences in the lifestyle of the participants. The participants in the study by Lee et al. (27) were collegiate athletes residing in on-campus dormitories and training locations and spent little time commuting to the campus. In contrast, the participants in this study lived individually in homes or apartments approximately 1 h from the campus, with their training sites also located away from the campus. As a result, this study's participants spent more time walking, running, and biking to school and their training locations, which may have resulted in a larger NEAT value than that in this prior study. Torstveit et al. (26) reported that some participants had physically active jobs, such as firefighting and carpentry, and that these athletes self-reported spending physically active leisure time, such as playing actively with their children, which could have contributed to the increase in NEAT volume. We found that the actual amount of NEAT was not constant, but it was widely distributed in both groups (Figure 2). Most variations in TEE that occur regardless of BW can be attributed to the variations in physical activity, with NEAT considered a significant contributor to inter- and intraindividual variations in energy expenditure (13). In addition, lifestyle and cultural milieu have been reported to be predictors of NEAT variability (29). In this context, athletes' NEAT is influenced by lifestyle and environmental factors in the same way as non-athletes.

To clarify the characteristics of NEAT, other energy components must be considered. In the present study, athletes had a significantly greater REE per day than non-athletes. Ratcliffe et al. (30) reported no significant differences in REE among resistance-trained men, endurance-trained men, and sedentary controls. The major determinant of REE is the FFM (31). FFM has the highest reported contribution to the REE in athletes (6). In this study, FFM was significantly higher in athletes than non-athletes (Table 1). Therefore, the higher REE per day in athletes may be due to the differences in FFM amounts resulting from variations in body size. DIT is influenced by the energy content and macronutrient composition (32), corresponding to 10% TEI in 24 h (9). TEI was significantly higher in athletes in this study. Thus, DIT was also higher in athletes. EEE is defined as the energy expended during the time spent in sports-specific training, warm-ups, and games (19). Therefore, given the selection criteria for participants in this study, higher EEE is natural.

NEAT and EEE accounted for 23.4% ± 3.3% and 14.3% ± 3.0% of TEE, respectively, with athletes' NEAT being approximately 1.7 times higher than their EEE in this study. High AEE, including NEAT and EEE, may induce an inappropriate energy balance and adversely affect physiological functions (33). Torstveit et al. (26) reported that NEAT tended to be higher in the group with suppressed REE. Although most studies have focused on EEE in energy expenditure (34), our study suggests that evaluating NEAT is necessary to assess energy expenditure in athletes. In support of our results, Taguchi and Manore (35) proposed using AEE, which includes not only EEE but also NEAT, to evaluate the physiologically available energy status. Future studies are required to investigate the relationship between NEAT and physiological functions.

In the present study, we aimed to characterize NEAT in athletes. To the best of our knowledge, no previous study has examined the activity intensity and duration of NEAT in athletes under real-life conditions. Several limitations need to be considered, including the small sample size and the restriction of sporting events to ball games. In addition, this study was restricted to male participants. NEAT is also influenced by occupation, body composition, and sex (13). Further studies with larger sample sizes and different types of sports, including women, should be conducted to characterize NEAT in athletes.

## 5 Conclusion

Overall, the results of the present study revealed that athletes do not expend more energy in NEAT relative to BW per day, but they spent less time on NEAT than non-athletes, which makes them more active in their daily lives, excluding exercise and sleep. NEAT accounted for about one-fourth of TEE in this study. Neglecting this parameter could lead to mistakes in the supervision of athletes. Therefore, assessing NEAT and EEE is necessary to determine the energy status of athletes.

## Data Availability

The datasets presented in this article are not readily available because of privacy reasons. Requests to access the datasets should be directed to MT, mtaguchi@waseda.jp.
